# Thrombocytopenia, Anasarca, Myelofibrosis, Renal dysfunction, and Organomegaly (TAFRO) Syndrome Initially Diagnosed as Fibromyalgia: A Case Report

**DOI:** 10.7759/cureus.42514

**Published:** 2023-07-26

**Authors:** Ryuichi Ohta, Chiaki Sano

**Affiliations:** 1 Community Care, Unnan City Hospital, Unnan, JPN; 2 Community Medicine Management, Shimane University Faculty of Medicine, Izumo, JPN

**Keywords:** japan, general medicine, atypical, community hospital, rural, tafro syndrome

## Abstract

Thrombocytopenia, Anasarca, myeloFibrosis, Renal dysfunction, and Organomegaly (TAFRO) syndrome is a rare and complex medical condition characterized by a combination of symptoms, including thrombocytopenia, anasarca, myelofibrosis, renal dysfunction, and organomegaly. The diagnosis of TAFRO syndrome can be challenging because of its rarity, overlapping symptoms, heterogeneity, and lack of specific biomarkers. We describe the case of a 77-year-old female who presented with fatigue and generalized arthralgia as chief complaints. Initially, the condition demonstrated no inflammatory manifestations for three months, and the patient was diagnosed with fibromyalgia. However, her symptoms progressed, and she eventually developed anasarca, thrombocytopenia, hepatosplenomegaly, and renal dysfunction. After using biopsy to exclude various diseases, we established a diagnosis of TAFRO syndrome and administered prednisolone and tocilizumab. The diagnosis was based on the clinical progression of anasarca, thrombocytopenia, hepatosplenomegaly, and renal dysfunction. To diagnose TAFRO syndrome, the intensive exclusion of various critical diseases is mandatory. Additionally, considering the gradual and fluctuating clinical course of TAFRO syndrome, physicians in rural areas should meticulously assess systemic symptoms in older patients.

## Introduction

Thrombocytopenia, Anasarca, myeloFibrosis, Renal dysfunction, and Organomegaly (TAFRO) syndrome is a rare and complex medical condition characterized by a combination of symptoms, including thrombocytopenia, anasarca, myelofibrosis, renal dysfunction, and organomegaly [1]. The diagnosis of TAFRO syndrome requires thorough clinical assessment. The initial step was to inquire about symptoms, including unexplained weight gain, swelling, fatigue, night sweats, fever, and general discomfort [2]. Thrombocytopenia is a hallmark laboratory feature of the TAFRO syndrome. Moreover, ultrasonography, computed tomography (CT), and magnetic resonance imaging (MRI) are techniques used to assess organomegaly, myelofibrosis, and lymphadenopathy. A tissue biopsy may be necessary to examine the affected organs or bone marrow for characteristic abnormalities [3]. Diagnosing TAFRO syndrome requires the exclusion of other potential causes of the symptoms, including lymphoma, Castleman disease, autoimmune disorders, and other hematological conditions. The diagnosis of TAFRO syndrome can be challenging because of its nonspecific symptoms [3,4].

Symptoms of the TAFRO syndrome can also be observed in other diseases, including lymphoma, Castleman disease, and autoimmune disorders. Moreover, TAFRO syndrome is a heterogeneous disorder with varied presentations and severity among individuals [5]. Currently, no specific biomarker can confirm TAFRO diagnosis. The diagnosis is primarily based on clinical features, laboratory tests, imaging studies, and other potential causes [6]. This report describes the case of a 77-year-old female with chief complaints of fatigue and systemic joint pain. The initial condition exhibited no inflammatory manifestations, and the patient was diagnosed with fibromyalgia. However, the symptoms progressed to anasarca, thrombocytopenia, hepatosplenomegaly, and renal dysfunction. After excluding various diseases using biopsies, we established the diagnosis of TAFRO syndrome and administered prednisolone and tocilizumab. The case report discusses the initial presentation of TAFRO syndrome along with practical diagnostic methods used in rural contexts.

## Case presentation

A 77-year-old female presented to a rural community hospital with chief complaints of fatigue and general arthralgia. Three months before admission, the patient experienced gradually worsening fatigue daily. Furthermore, systemic joint pain gradually extended to the hands and feet two months before admission. One month before admission, fatigue, and joint pain aggravated, causing loss of appetite. One week before admission, the patient could not perform daily activities while living independently, such as eating and using the toilet. The patient visited our hospital, and we began investigating her symptoms, including weight loss and night sweats. She had no organ-specific symptoms such as rash, dry mouth, dry eye, headache, cough, chest pain, abdominal pain, vomiting, or diarrhea. Her medical history included osteoporosis and depression, and she received 20 mg of paroxetine daily.

The vital signs at the initial visit were: 124/76 mmHg blood pressure, 77 beats/min pulse rate, 35.2 °C body temperature, 20 breaths/min respiratory rate, and 96% room air oxygen saturation. The patient was alert to time, place, and person. Physical examination revealed joint tenderness in her bilateral hands, shoulders, thighs, feet, and neck without swelling or erythema. Lower extremity examination revealed bilateral pitting edema. The patient’s oral cavity had dry mucous membranes. No other neurological abnormalities were observed, including the absence of pallor, icterus, apparent chest, abdominal, or skin abnormalities. Laboratory tests revealed mild inflammation with C-reactive protein (CRP) elevation, mild anemia, severe hyponatremia, and hypoalbuminemia (Table 1).

**Table 1:** Initial laboratory data of the patient eGFR, estimated glomerular filtration rate; CK, creatine kinase; CRP, C-reactive protein; TSH, thyroid-stimulating hormone; Ig, immunoglobulin; SARS-CoV-2, severe acute respiratory syndrome coronavirus 2; C3, complement component 3; C4, complement component 4; MPO-ANCA, myeloperoxidase antineutrophil cytoplasmic antibody; CCP, cyclic citrullinated peptide

Parameters	Level	Reference
White blood cells	4.90	3.5–9.1 × 10^3^/μL
Neutrophils	86.1	44.0–72.0%
Lymphocytes	5.4	18.0–59.0%
Monocytes	7.9	0.0–12.0%
Eosinophils	0.1	0.0–10.0%
Basophils	0.5	0.0–3.0%
Red blood cells	3.18	3.76–5.50 × 10^6^/μL
Hemoglobin	9.8	11.3–15.2 g/dL
Hematocrit	29.9	33.4–44.9%
Mean corpuscular volume	94.0	79.0–100.0 fL
Platelets	21.7	13.0–36.9 × 10^4^/μL
Erythrocyte sedimentation rate	56	2–10 mm/h
Total protein	6.9	6.5–8.3 g/dL
Albumin	3.3	3.8–5.3 g/dL
Total bilirubin	0.9	0.2–1.2 mg/dL
Aspartate aminotransferase	26	8–38 IU/L
Alanine aminotransferase	20	4–43 IU/L
Alkaline phosphatase	181	106–322 U/L
γ-Glutamyl transpeptidase	16	<48 IU/L
Lactate dehydrogenase	160	121–245 U/L
Blood urea nitrogen	22.0	8–20 mg/dL
Creatinine	0.59	0.40–1.10 mg/dL
eGFR	73.6	>60.0 mL/min/L
Serum Na	121	135–150 mEq/L
Serum K	4.0	3.5–5.3 mEq/L
Serum Cl	81	98–110 mEq/L
Serum Ca	9.1	8.8–10.2 mg/dL
Serum P	3.3	2.7–4.6 mg/dL
Serum Mg	2.1	1.8–2.3 mg/dL
Serum iron	54	43–172 μg/dl
Ferritin	322.8	14.4–303.7 ng/mL
CK	120	56–244 U/L
CRP	4.06	< 0.30 mg/dL
TSH	2.60	0.35–4.94 μIU/mL
Free T4	1.4	0.70–1.48 ng/dL
IgG	1218	870–1700 mg/dL
IgM	176	35–220 mg/dL
IgA	231	110–410 mg/dL
IgE	28	< 173 mg/dL
SARS-CoV-2 antigen	-	
anti-nuclear antibody	40	< 40
C3	105	86–164 mg/dL
C4	27	17–45 mg/dL
MPO-ANCA	< 1.0	< 3.5 U/mL
anti-CCP antibody	< 0.6	< 5 U/mL
Soluble interleukin 2 receptor	393	122–496 U/mL
Urine test		
Leukocyte	Negative	Negative
Nitrite	Negative	Negative
Protein	Negative	Negative
Glucose	Negative	Negative
Urobilinogen	normal	
Bilirubin	Negative	Negative
Ketone	Negative	Negative
Blood	Negative	Negative
pH	7.0	

Therefore, we suspected hyponatremia with systemic disease. Serum iron levels were within the normal range, whereas ferritin levels were elevated, indicating chronic inflammation. Normal random and adrenocorticotropic hormone stimulation tests revealed normal cortisol secretion, ruling out adrenal insufficiency. However, the antidiuretic hormone levels were low, indicating the possibility of mineralocorticoid-responsive hyponatremia in older individuals. Subsequently, the patient was treated daily with 15 mg of hydrocortisone. 

On day 7, the patient developed a mild fever of 37.3 °C, and systemic edema appeared with mild dyspnea. Chest radiography revealed bilateral infiltration at the dull costophrenic angle. Urine analysis revealed white blood cells and organisms, and Gram staining demonstrated Gram-negative rods and white blood cells, suggesting a urinary tract infection. Considering the clinical course, we established a diagnosis of a urinary tract infection with congestive heart failure and administered furosemide (20 mg/day) and ceftriaxone (2 g/day) for one week. Subsequently, the dyspnea and systemic edema disappeared.

On day 21, systemic joint pain persisted, with no inflammation in the laboratory data, including non-significant results for CRP and erythrocyte sedimentation rate (ESR). All affected joint ultrasonography revealed no signs of inflammation. Based on the chronic clinical course of the systemic pain, the patient was diagnosed with fibromyalgia based on the American College of Rheumatology and received daily treatment of 500 mg acetaminophen and 10 mg paroxetine. The treatments above were partially effective, and the systemic joint pain was gradually alleviated.

**Figure 1:**
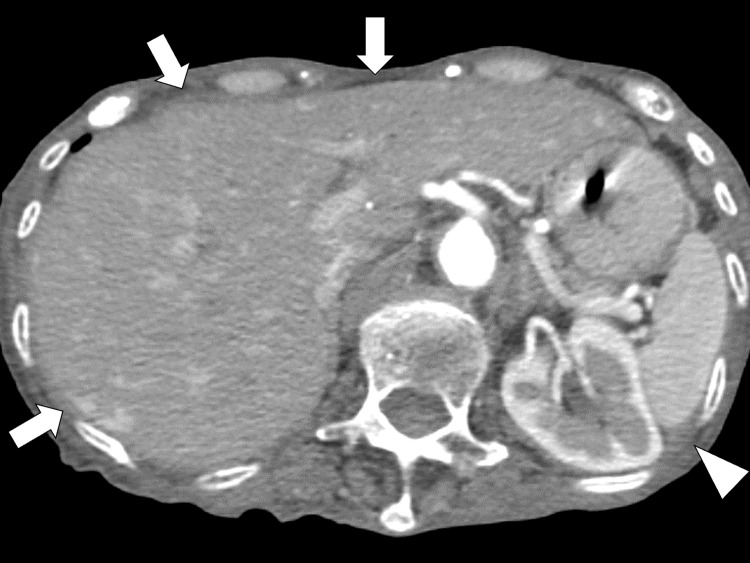
An enhanced neck-to-pelvic computed tomography scan The image is revealing pleural effusion, ascites (white arrowhead), and hepatosplenomegaly (white arrow) without prominent lymphadenopathy.

On day 30, the patient developed a 38.5 °C fever and a gradually worsening systemic edema. Additionally, she had a decreased appetite and dry oral mucous membranes. Laboratory data revealed thrombocytopenia of 4.1 × 10^4^/μL, renal dysfunction of 32 mL/min/L, alkaline phosphatase of 261 U/L, interleukin (IL)-6 of 28.1 pg/mL (normal range: <10 pg/mL), as well as elevated inflammatory markers of CRP and ESR of 6.4 mg/dL and 41 mm/h, respectively. No coagulopathy was in the laboratory, and urine analysis revealed no evidence of pyuria. Enhanced neck-to-pelvic CT revealed bilateral pleural effusion, ascites, and hepatosplenomegaly without prominent lymphadenopathy (Figure 1).

Random skin and lip biopsies were performed to exclude intravascular lymphoma and investigate the symptoms. We could not perform a bone marrow biopsy because of the patient's refusal. On day 33, the blood, sputum, and urine culture results were negative. A lumbar puncture was performed to exclude meningitis, which revealed no elevation of proteins, cells, or IL-6 in cerebrospinal fluid. As acetaminophen and paroxetine were suspected to cause drug fever, the medications were discontinued. Additionally, peroxidase and protease-3 anti-neutrophil cytoplasmic antibodies were assessed to investigate vasculitis, and the results were negative.

Based on the clinical course and negative studies, the patient was diagnosed with TAFRO syndrome and was administered 1 mg/kg prednisolone daily and 162 mg tocilizumab weekly. Platelet counts increased, and ascites and pleural effusion decreased. Within a week, the patient’s appetite increased. However, three weeks after initiating treatment, the patient became unconscious with a 39.4 °C fever, tachypnea, tachycardia, and hypotension. As no sputum, pyuria, or apparent etiologies were present, the patient was diagnosed with septic shock due to bacterial translocation in the gastrointestinal tract caused by her immunosuppressive condition. She was administered 3 g of meropenem and 2 g of vancomycin daily. Unfortunately, the patient succumbed two days later due to cardiorespiratory arrest from septic shock.

## Discussion

The case report describes atypical TAFRO syndrome without initial inflammatory manifestations that were transiently diagnosed as fibromyalgia. The progression of the clinical course to anasarca, thrombocytopenia, hepatosplenomegaly, and renal dysfunction enabled us to establish a correct diagnosis. To diagnose TAFRO syndrome, physicians must intensively exclude various critical diseases and consider the gradual and fluctuating clinical courses of TAFRO syndrome. Furthermore, rural physicians should meticulously assess systemic symptoms in older adults considering the possibility of TAFRO syndrome and ruling out various critical diseases. 

An atypical clinical course of TAFRO syndrome should be considered in patients with unknown inflammatory conditions after ruling out infection, lymphoma, and vasculitis. The typical clinical course of TAFRO syndrome involves the rapid onset of symptoms, often leading to hospitalization, and such patients commonly present with fatigue, fever, weight loss, anasarctic edema, and enlarged lymph nodes. Additionally, thrombocytopenia, myelofibrosis, renal dysfunction, and hepatomegaly/splenomegaly are also frequently observed. Regarding the atypical clinical course, variations in the initial presentation and progression of TAFRO syndrome have been reported [7]. Moreover, some patients may initially present with mild symptoms or may not encompass the features of all five syndromes [8]. In such cases, the diagnosis may be more challenging and delayed. Additionally, the disease progression rate and treatment response varies among individuals. Some patients experience a rapidly progressive disease course requiring immediate interventions, including immunosuppressive therapy, whereas others may experience a more indolent disease course [9]. Relapsed or refractory disease cases have been reported in which patients experience recurrent symptoms or have an inadequate initial treatment response [10,11]. These patients may require aggressive therapy or alternative treatment strategies. Given the rarity of the TAFRO syndrome and the limited number of reported cases, its atypical clinical course remains largely unknown. Further research and clinical studies of this complex syndrome are necessary to enhance our understanding of its presentation and outcomes.

Family physicians practicing in rural contexts should be educated about the TAFRO syndrome and its various presentations. Diagnosing TAFRO syndrome in rural areas can be challenging owing to the complexity of the condition and the limited availability of healthcare resources [12]. As the report revealed, diagnosing TAFRO syndrome in older patients is challenging because symptoms are vague and sometimes progressive and intermittent, which can delay the decision for further investigation and consultation with multiple professionals. The diagnosis mimics other disease processes contributing to confusion and delayed diagnosis. Bone marrow and additional tissue biopsy may be necessary to confirm the diagnosis and exclude other conditions [13], as it can reveal characteristic histological features, including vascular proliferation, fibrosis, and inflammatory infiltrates [14]. However, it is essential to consider organ accessibility when performing and analyzing biopsies in rural areas. In our patient, we performed a biopsy based on skin and lip symptoms. TAFRO syndrome may not demonstrate prominent lymphadenopathy; thus, rural family physicians should consider this possibility and investigate realistic methods for diagnosing and excluding other critical diseases.

Diagnosing TAFRO syndrome based on its gradual and fluctuating clinical course can be challenging, given the varying presentations and progression of symptoms. As this case demonstrates, TAFRO syndrome can have an inconsistent clinical course, although previous studies have revealed that TAFRO syndrome exhibits an acute and progressive clinical course. Patients with TAFRO syndrome may show a fluctuating clinical course with a non-inflammatory interval and may experience intermittent symptom exacerbation and remission [15]. Documenting the specific symptoms experienced by patients is essential and may include fatigue, fever, weight loss, edema, and organomegaly. Diagnosing TAFRO syndrome based on its gradual and fluctuating clinical course may require longitudinal assessment over an extended period [16]. Repeated evaluations, including clinical examinations, laboratory tests, and imaging studies, may be necessary to establish symptom patterns and document the progression or changes in disease activity. As system-specific specialists, family physicians should use their collective expertise to interpret and integrate information from various diagnostic modalities to reach a comprehensive diagnosis of TAFRO syndrome [17,18].

## Conclusions

This case report describes the atypical presentation of TAFRO syndrome without initial inflammatory manifestations, resulting in a transient diagnosis of fibromyalgia. A correct diagnosis was established because of the progressive clinical course of the disease with anasarca, thrombocytopenia, hepatosplenomegaly, and renal dysfunction. When diagnosing TAFRO syndrome, intensive exclusion of various critical diseases is mandatory, considering its gradual and shifting clinical course. Furthermore, rural physicians should meticulously assess the systemic symptoms of older adults and investigate them comprehensively considering lymphoproliferative diseases such as TAFRO syndrome.
